# Supplementary material for: A set of multi-entry identification keys to African frugivorous flies (Diptera, Tephritidae)
Source: Zookeys. 2014 Jul 24;(428):97–108. doi: 10.3897/zookeys.428.7366 (PMC4143993; doi:10.3897/zookeys.428.7366)
Supplement: Supplementary material 4 — Key to Capparimyia [file zookeys-428-097-s004.zip › SF4_ZooKeys_key to Capparimyia/lucid_player/help/help/java_-_what_does_it_mean_.htm]

Java - What does it mean?


# Java - What does it mean?

Lucid3 has been developed using the Java platform. This allows Lucid3
to run under any operating system that supports the Java Runtime Environment
(JRE) including Microsoft Windows1,
Mac OSX, Unix, Linux and Sun Solaris.

For Lucid3 to install and run on your computer you need to ensure you
have the latest Java Virtual Machine2
(JVM) installed. Windows users can find a copy of the JVM on the Lucid3
CD. Other operating system owners either already have the JVM as part
of their operating system or can obtain it through their system update
utilities. It is recommended however that you check www.java.com
for the latest edition (available free of charge). Mac users should check
with www.apple.com/java

For more information on Lucid3 and Java please see the FAQ topic www.lucidcentral.org/support/faq\_lucid3.htm

1 Please note that Sun,
the maker of Java, does not support Windows 95, hence Lucid3 does not
operate on Windows 95.

2 The Java Runtime Environment
is sometimes also referred to as the Java Virtual Machine (JVM). This
is actually a subset of the JRE, but the two terms are often used interchangeably.
